# Development of Human iPSC-based Microphysiogical Models of Transthyretin Amyloid Cardiomyopathy

**DOI:** 10.21203/rs.3.rs-9636540/v1

**Published:** 2026-06-10

**Authors:** Marcus A.C. Williams, Junseong Park, Atharva R. Mulay, Devin Mair, Colton R Lysaker, Vivek Jani, Desirae McKoy, Marcus Rhodehamel, Parisha Garg, Alex Wu, Sangeeta Koilada, Sharanya Parvathaneni, Brian Lin, Heather M. Wilkins, Kavita Sharma, Joban Vaishnav, Mark J. Ranek

**Affiliations:** The Johns Hopkins Medical Institutions; The Johns Hopkins Medical Institutions; The Johns Hopkins Medical Institutions; The Johns Hopkins Medical Institutions; University of Kansas Medical Center; The Johns Hopkins Medical Institutions; The Johns Hopkins Medical Institutions; The Johns Hopkins Medical Institutions; The Johns Hopkins Medical Institutions; The Johns Hopkins Medical Institutions; The Johns Hopkins Medical Institutions; The Johns Hopkins Medical Institutions; The Johns Hopkins Medical Institutions; University of Kansas Medical Center; The Johns Hopkins Medical Institutions; The Johns Hopkins Medical Institutions; The Johns Hopkins Medical Institutions

**Keywords:** Transthyretin, cardiac, amyloidosis, in vitro, iPSCs

## Abstract

Transthyretin (TTR) amyloid cardiomyopathy (ATTR-CM) is a restrictive cardiac disease caused by the deposition of TTR in the heart. TTR is synthesized by hepatocytes and circulates as a homotetramer carrying complex for thyroxine and retinol. Mutations in TTR destabilize the tetramer promoting its dissociation into monomers that can enter the heart and aggregate into amyloid fibrils driving cytotoxicity, fibrosis, impaired electrical conductivity, and progressive cardiac dysfunction. Mechanistic studies and therapeutic development have been limited by the lack of physiologically relevant human preclinical models. Here we describe the creation of two *in vitro* models of ATTR-CM using human iPSC-derived cells. First, a custom microphysiological chip containing co-cultured hepatic and cardiac organoids in isolated chambers connected by microchannels enabling free passage, including hepatocyte synthesized TTR^122VI^, which diffuses to and is taken up by cardiac organoids recapitulating pathologic hepatic-cardiac crosstalk. Cardiac organoids exposed to TTR^V122I^ exhibited increased oxidative stress, elevated cytotoxicity, and upregulation of fibronectin. Second, engineered heart tissues cultured with monomeric TTR^V122I^ demonstrated TTR uptake, reduced contractility, and prolonged relaxation time that mirrors cardiac dysfunction observed clinically. These novel human-based models recapitulate key biochemical, cellular, and functional features of ATTR-CM, providing translational platforms to elucidate disease mechanisms and evaluate novel therapeutic strategies.

## Introduction

Transthyretin amyloid cardiomyopathy (ATTR-CM) is a form of restrictive cardiomyopathy characterized by the deposition of misfolded transthyretin (TTR) amyloid fibrils within the myocardium, leading to increased wall stiffness, impaired diastolic relaxation, and eventual heart failure.^1^ Despite growing clinical awareness, ATTR-CM remains substantially underdiagnosed. A recent international study identified that approximately 18% of individuals diagnosed with heart failure with preserved or mildly reduced ejection fraction were confirmed for ATTR-CM by cardiac scintigraphy despite no prior diagnosis of amyloidosis.^2^ ATTR-CM impacts an estimated 120,000 individuals in the United States with numbers continuing to rise with improvement in detection and diagnostic imaging.^3–5^ ATTR-CM manifests through two distinct mechanisms: a hereditary or variant form (ATTRv-CM) that is driven by mutations in the TTR gene causing destabilization of the native homotetrameric complex thereby promoting amyloid formation, and a wild-type form (ATTRwt-CM) where the protein idiopathically dissociates over time predominantly presenting in older individuals.^1,3^ TTR is primarily synthesized in the liver and secreted into circulation as a homotetramer that functions as a transport protein for thyroxine (T4) and retinol (vitamin A) via retinol-binding protein. Disruption of the tetramer conformation leads to monomeric dissociation which can aggregate into amyloid fibrils that can deposit in the myocardium causing cytotoxicity, extracellular stiffening, enhanced fibrosis, and progressive cardiac dysfunction.^1,6^ Among pathogenic variants of TTR, the valine-to-isoleucine substitution at position 122 (V122I) is the most clinically pathogenic and pervasive in the United States affecting approximately 4% of Black Americans.^7^ Despite the growing burden of disease, the molecular and cellular mechanisms by which TTR deposition drives myocardium dysfunction remain incompletely understood.

The past decade has seen the emergence of several disease-modifying therapies for ATTR-CM with demonstrated efficacy at slowing disease progression and improving quality of life. These include TTR stabilizers (e.g. tafamidis and acoramidis), which stabilize the TTR complex thereby preventing dissociation, and TTR gene silencers (e.g. patisiran and inotersen), which reduce hepatic TTR production through RNA interference and antisense mechanisms, respectively.^6,8–10^ While these therapies delay disease progression, they share fundamental limitations as they target TTR at either the level of production or stabilization of the tetramer but do not address downstream cardiac impairment from amyloid deposition. Many individuals do not receive treatment until pathological symptoms present making progression towards ATTR-CM mediated heart failure a major clinical challenge as no currently available therapy has been shown to rescue cardiac dysfunction.^8^ Patients with advanced ATTR-CM are frequently intolerant of conventional heart failure therapies, and mechanisms of pathological remodeling that impairs cardiac function remain poorly characterized.^1,11^ This therapeutic gap underscores the pressing need to establish greater mechanistic insight into the cardiac pathology of ATTR-CM and for the creation of novel model systems that can support the development of cardiac-directed therapeutic strategies.

Investigations into ATTR-CM pathogenesis and evaluation of novel therapeutics, particularly for the heart, have been significantly limited by the lack of preclinical models that recapitulate the physiological features of the disease (e.g. hepatic expression and cardiac uptake). Various transgenic mouse models have been generated expressing mouse of human TTR, but these have been largely unsuccessful due to inconsistent or absent cardiac phenotypes, prolonged aging required for amyloid deposition, and fundamental interspecies differences that limit translational relevance.^12,13^ In vitro models have been more successful by subjecting cardiomyocytes to fibril conditioned media,^14–16^ direct incubation with recombinant TTR protein,^17^ or culturing cardiomyocytes on TTR fibril-coated substrates.^13,18^ While these systems have provided valuable insight into TTR-mediated cardiotoxicity through demonstrating increased oxidative stress, protein aggregation, and cell death, they possess several key limitations. First, they rely on exogenous application of TTR or manual media transfer rather than continuous physiological secretion, and therefore, do not capture the hepatic-cardiac crosstalk that defines ATTR-CM pathophysiology. Second, most are limited to 2D monolayer cultures that lack 3D tissue architecture. Third, and most critically, none enable functional readout of tissue-level cardiac mechanical performance despite contractile dysfunction and impaired relaxation being hallmark clinical features of the disease.^13^

To address these gaps, we developed two complementary 3D in vitro models of ATTR-CM engineered with human induced pluripotent stem cells (iPSCs) that recapitulate key morphological, biochemical, and physiological features of the disease. These models enable investigation of the molecular, cellular, and functional mechanisms underlying disease pathogenesis as well as evaluation of preclinical therapeutic strategies. The first model utilizes a custom microfluidic chip platform loaded with 3D cardiac and hepatic organoids generated from human iPSC-derived cardiomyocytes (hiPSC-CMs) and hepatocytes (hiPSC-HTs) harboring the TTR^V122I^ mutation. These organoids are cultured in physically separate wells connected by microchannels that inhibit cellular migration while permitting paracrine crosstalk. Mutant TTR^V122I^ is synthesized and secreted by the hepatic organoid, enabling subsequent uptake by the cardiac organoid, thereby emulating the microcirculatory environment and the cardiac-hepatic axis central to ATTR-CM pathogenesis. The second model consists of engineered heart tissues (EHTs) composed of hiPSC-CMs and human cardiac fibroblasts (HCFs) functionalized on a mechanically loaded platform enabling direct measurement of contractile force and relaxation kinetics after exposure to monomeric mutant TTR that mimic circulating physiological levels.^19,20^

Cardiac organoids co-cultured with TTR^V122I^-expressing hepatic organoids demonstrated TTR uptake and subsequently increased oxidative stress, elevated cleaved caspase-3, and upregulation of fibrotic markers. EHTs cultured with exogenous mutant monomeric TTR similarly demonstrated uptake and developed reduced peak contractile force and prolonged relaxation time thereby mirroring the systolic and diastolic dysfunction observed clinically in disease. Together, these human-based in-vitro models capture the central biochemical, cellular, and functional features of ATTR-CM, providing physiologically relevant platforms to elucidate disease mechanisms and evaluate novel therapeutic strategies that directly target cardiac function.

## Methods

### Differentiation of hiPSCs into cardiomyocytes

Human induced pluripotent stem cells (hiPSCs; line JHU001, healthy donor) were obtained from Johns Hopkins University.^21–23^ hiPSCs were cultured in Essential 8 media (ThermoFisher Scientific, Cat. #A1517001) and differentiated into cardiomyocytes using established protocols.^24,25^ On day (−3), T75 flasks were coated with 10 ml of Geltrex (Gibco) solution (1:200 in RPMI 1640) and allowed to incubate at 37°C for 1 hour prior to use. Post coating, 5.0 × 10^5 hiPSCs were seeded onto the T75 flasks and cultured in 10 ml of Essential 8 media with Y27632 (ROCK inhibitor, Tocris, Cat. #1254, 1:1000) for 24 hours. After 24 hours the media was exchanged daily with 20 ml of Essential 8 without ROCK inhibitor Y27632 and the cells were cultured until 95% confluency. On day 0, iPSCs were exposed to CHIR99021 (GSK-3β inhibitor/Wnt activator, Tocris, Cat. #TB4423-GMP; 6 μM) in RPMI1640 supplemented with B-27 minus insulin, (ThermoFisher Scientific, Cat. #A1895602) for 48 hours to induce differentiation into mesodermal cells. On day 2, the media was replaced with RPMI1640/B27 minus insulin for 24 hours. On day 3, IWR-1 (Wnt inhibitor, Tocris, Cat. #3532; 5 μM) was added to RPMI1640 supplemented with B-27 minus insulin for 48 hours to promote cardiomyocyte differentiation. Thereafter, media was exchanged every 48 hours with RPMI1640 supplemented with B-27 minus insulin until day 8. By day 9, cardiomyocytes were beating and cultures maintained in RPMI 1640 with B-27 supplement with insulin and without serum (ThermoFisher Scientific, Cat. #11875093 and 17504044, respectively). Fresh media was added every 2 days. On day 13, cardiomyocytes were replated into two T75 flasks with RPMI1640/B-27 with Y27632 for 24 hours, followed by two days in RPMI1640/B-27 without ROCK inhibition. Lactate selection was then performed using lactate-containing selection media (ThermoFisher Scientific, with sodium lactate, 4 mM) for 2 days to enrich cardiomyocytes, after which cultures were returned to RPMI 1640 with B-27 supplement without serum.

### Differentiation of hiPSCs into hepatocytes

JHU001 hiPSCs were maintained in Essential 8 media and differentiated into hepatocytes by using published protocols.^26^ hiPSCs were seeded at 2.5 × 10^5 cells per well in a 6 well plate with Y27632 (1:1000) for 24 hours. Definitive endoderm (DE) induction was carried out for 3 days in RPMI1640 media with B-27 supplement containing Activin A (Thermo Fisher Scientific, Cat. #PHG9014, 100 ng/ml) and CHIR99021 (Tocris, 3 μM). Cells were then transitioned to hepatic endoderm (HE) media containing BMP4 (Sigma Aldrich, Cat. #H4916, 20 ng/ml), bFGF (R&D Systems, Cat. #233-FB-025/CF, 5 ng/ml) and DMSO (Sigma-Aldrich, Cat. #151874-50G-SB, 0.5%) for 5 days. Immature hepatocytes were further cultured for 5 days in intermediate hepatocyte (IMH) media containing hepatocyte growth factor (HGF) (Sigma Aldrich, Cat. #H9661, 20 ng/ml) and DMSO (5%). Cells were then matured for 10 days in mature hepatocyte (MH) media (hepatocyte basal media supplemented with SingleQuots (Lonza, Cat. #CC-3198), containing HGF (Sigma Aldrich, Cat. #H9661-5UG, 20 ng/ml), Oncostatin M (Stemcell Technologies, Cat. #78094, 20 ng/ml), dexamethasone (Millipore Sigma, Cat. #D4902-100MG, 100 nM), and DMSO (0.5%). Cells were collected for hepatocyte lineage phenotypic characterization and organoid formation.

### Generation of 3D cardiac and hepatic organoids

Organoids were generated using the AggreWell^™^ 800 protocol (Stem Cell Technologies, Cat. #34850) per manufacturer’s instructions. Briefly, plates were pretreated with anti-adherence rinsing solution to promote efficient formation of embryoid bodies (EBs) or spheroids. For cardiac organoids, a single-cell suspension of 70% hiPSC-derived cardiomyocytes and 30% human cardiac fibroblasts (HCF) was prepared; for hepatic organoids, a suspension of hiPSC-HTs was used. A total of 2.4 × 10^5 cells per well were seeded into AggreWell^™^ 800 plates, distributed evenly, and centrifuged to capture cells in microwells. Organoids formed within 24 to 48 hours. Organoids were maintained in RPMI 1640/B27 media with changes occurring as needed and harvested per manufacturer’s guidelines.

### Fabrication of a custom microfluidic chip

Microfluidic devices with two chambers connected by microchannels were designed using computer-aided design (CAD) software. Devices were fabricated in polydimethylsiloxane (PDMS, Sylgard 184, Dow Corning) for its optical clarity, elasticity, low toxicity, chemical inertness, affordability, and gas permeability.^27^ PDMS base and curing agent were mixed (10:1), degassed, poured over the custom patterned mold printed by Protolabs using our design, and cured at 60°C for 24 hours. PDMS slabs were peeled and bonded to glass slides using oxygen plasma treatment (FEMTO low-pressure plasma system, Diener electronic GmbH + Co. KG) at 150 watts for 90 seconds, then baked in an oven at 60°C overnight to strengthen bonds and complete the PDMS curing process. Devices were cleaned and sterilized in preparation for organoid loading. Organoids were introduced into the respective chambers and device ports were opened/closed as required for experimental procedures.

### Generation of engineered heart tissues (EHT)

EHTs were generated using the MyoPod system (Propria LLC) according to the manufacturer's instructions. Briefly, MyoPod scaffolds were rinsed with RPMI 1640 supplemented with B27 to remove the preservation solution. hiPSC-CMs and HCFs were dissociated using Accutase and passed through a 100 μm cell strainer to obtain single-cell suspensions. Cells were combined at a ratio of 10 hiPSC-CMs (2.0 × 10^6^) to 1 HCF (0.2 × 10^6^) at a density of 2.2 × 10^6^ total cells per scaffold and seeded to form EHTs. After 24 hours, scaffolds were transferred to 12-well plates and maintained in RPMI 1640/B27 media, with exchanges every 48 hours for 3 weeks. TTR was amplified, isolated, and purified according to manufacturer’s protocol (ProMab).

### Immunofluorescence staining

Organoids and EHTs were washed with PBS (3x) to remove excess media and fixed in 4% paraformaldehyde at 4°C for 2 hours. After fixation, organoids were incubated in blocking buffer (30 ml PBS, 3 ml FBS, 60 μL Triton X100) for 1 hour at 4°C. Organoids were incubated overnight at 4°C with primary antibodies against cardiac troponin T (cTnT, Abcam; Cat. #ab8295, Abcam, 1:100) and transthyretin (Abcam; Cat. #ab75815, 1:200) prepared in antibody diluent buffer. Following three washes with blocking buffer, organoids were incubated overnight at 4°C with Alexa Fluor-conjugated secondary antibodies (ThermoFisher Scientific, Alexa 488 donkey anti-rabbit IgG and Alexa 568 goat anti-mouse). The next day, organoids were washed with blocking buffer, followed by rinse buffer (30 ml PBS, 60 ml FBS, 60 μL Triton X100). Organoids were transferred to glass-bottom dishes, extra buffer was removed, and samples were mounted with Vectashield mounting agent with DAPI (Vector Laboratories, Cat. #H-2000-2). Samples were imaged using confocal microscopy (Zeiss 710NLO-Meta).

### Western blotting

Organoids and tissues were lysed in 1X RIPA buffer containing protease and phosphatase inhibitor (Cell Signaling Technology). Protein concentration was measured by a bicinchoninic acid (BCA) assay (Pierce), and extracts were subjected to Western Blot (Bio-Rad). Whole cell lysate (25μg) was loaded on the BioRad gels (TGX 4–20%) and transferred to nitrocellulose membranes. Membranes were blocked for one hour in blocking buffer (LiCor) and incubated overnight in 4°C with primary antibodies transthyretin (Abcam, Cat. #ab271132, 1:1000), cTnT (Abcam, Cat. #ab115234, 1:1000), cleaved caspase-3 (Abcam, Cat. #ab32042), and albumin (Abcam, Cat. #ab207327, 1:1000) in antibody diluent buffer (LiCor). Membranes were washed in TBS-T and incubated with IRDye secondary antibodies anti-mouse or anti-rabbit (1:10,000) in antibody diluent buffer for one hour in room temperature. Western blots were visualized using Li-Cor Odyssey imaging system (Odyssey CLx) and quantified in ImageJ (NIH). Signal normalization was performed to total protein stain (Li-Cor).

### Quantitative polymerase chain reaction (qPCR)

Total RNA was extracted from hiPSC-HTs and iPSCs (controls) using Trizol (Invitrogen) per manufacturer’s instructions. RNA purity and concentration were assessed prior to cDNA synthesis. cDNA was generated using a high-capacity RNA-to-cDNA kit (Applied Biosystems). Quantitative PCR was performed using TaqMan assays (Applied Biosystems) for TTR (ThermoFisher, Cat. #4331182, Hs00174914_m1) ASGR1 (ThermoFisher, Cat. #4331182, Hs01005019_m1), HNF4 alpha (ThermoFisher, Cat. #4331182, Hs00230853_m1), FN1 (ThermoFisher, Cat. #4331182, Hs00415006_m1), CYP3A4 (ThermoFisher, Cat. #4331182, Hs00604506_m1), and Albumin (ThermoFisher, Cat. #4331182, Hs00609411_m1) with GAPDH (ThermoFisher, Cat. #4331182, Hs02786624_g1) as the control. Reactions were run on a real-time PCR machine (Quantstudio), with three technical replicates per sample. Ct values were determined by the crossing point method.

### Contractile Function Measurement Protocol

EHT contractile performance was assessed using the MyoLab platform according to the manufacturer’s protocol. Briefly, tissues were equilibrated at 37°C in the MyoLab measurement chamber. Spontaneous contractions were recorded without external electrical pacing to capture intrinsic biomechanical properties. Systolic and diastolic functional metrics were then recorded, extracted, and analyzed.

### CellROX Staining

CellROX reagent (ThermoFisher, Cat. #C10444) was used at a final concentration of 5 μM. Organoids were incubated at 37°C for 30 minutes with the CellROX reagent. The samples were then briefly washed with PBS 3x, fixed, and imaged on a confocal microscope.

#### Bioethical statement

The authors declare no competing interests. The protocols used in this study were approved by Johns Hopkins University and conducted in accordance with the guidelines and regulations set forth by the institution. The cell line was derived from an adult who signed an informed consent form and has been previously published.^21–23^

## Results

### Design and fabrication of a microphysiological device to model ATTR-CM

**Figure 1:**
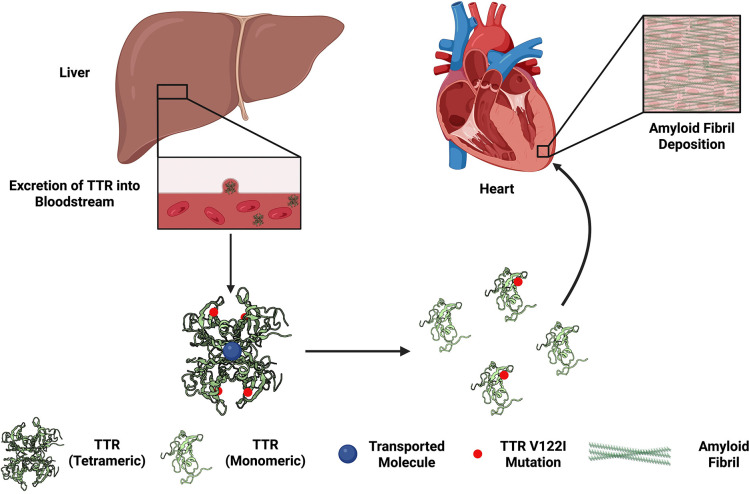
Overview of the development of hereditary transthyretin cardiac amyloidosis. The liver synthesizes and secretes transthyretin (TTR) into the circulation to function as a transport protein complex. Mutations in TTR (e.g. V122I) increase the propensity of the TTR tetrameric complex to dissociate into monomers. TTR monomers can be taken up by the myocardium, aggregated, and form amyloid fibrils. The deposition of amyloid fibrils in the heart may lead to restrictive cardiomyopathy. Created in BioRender. Ranek, M. (2026) https://BioRender.com/3saqgmj.

**Figure 2:**
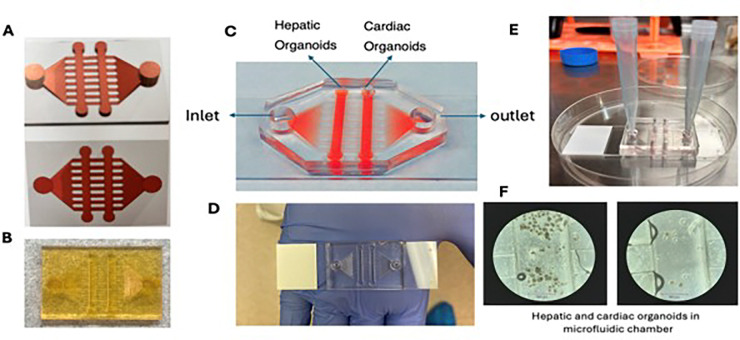
Microfluidic chip design and formation. **A)** Solidworks CAD file for the negative mold utilized for fabrication of the PDMS microfluidics device. **B)** Stereolithography 3D printed mold. **C)** Functional PDMS based microfluidics device capable of both passive and active diffusion. **D)** PDMS mold plasma bonded onto the slide to form the chip. **E.)** Functional prototype of loading the organoids into the chip. **F)** Images of organoids inside chambers of the microfluidic chip.

An overview of ATTR-CM pathogenesis is illustrated in Fig. 1. To model the dynamics of inter-organ crosstalk in ATTR-CM, we developed a two-chamber microfluidic platform designed for the co-culture of hiPSC-derived hepatic and cardiac organoids in physically isolated environments. Interconnected microchannels were incorporated to enable paracrine secretion and signaling from adjacent organoids mimicking hepatic-cardiac axis in ATTR-CM. The device was designed using computer-aided design software (Fig. 2A) and a master mold was fabricated via stereolithography 3D printing for reproducible manufacturing (Fig. 2B). Polydimethylsiloxane (PDMS) was selected as the device material for its optical transparency, elasticity, and biocompatibility^27–29^ and was cast into the mold to produce chips with dedicated organoid chambers and interconnecting microchannels that permit soluble factor exchange, thereby simulating microcirculatory transport while preventing cellular migration (Fig. 2C). After curing, the PDMS device was plasma bonded to a glass slide to seal the channels and complete the assembly (Fig. 2D). Prior to use, chips were sterilized, washed, and primed with culture media to promote surface wetting and remove residual uncured polymer. Organoids were loaded into their respective chambers, and routine media exchanges were performed through designated inlets and outlets to support long-term viability and function (Fig. 2E–G).

### Characterization of hepatic organoids

**Figure 3:**
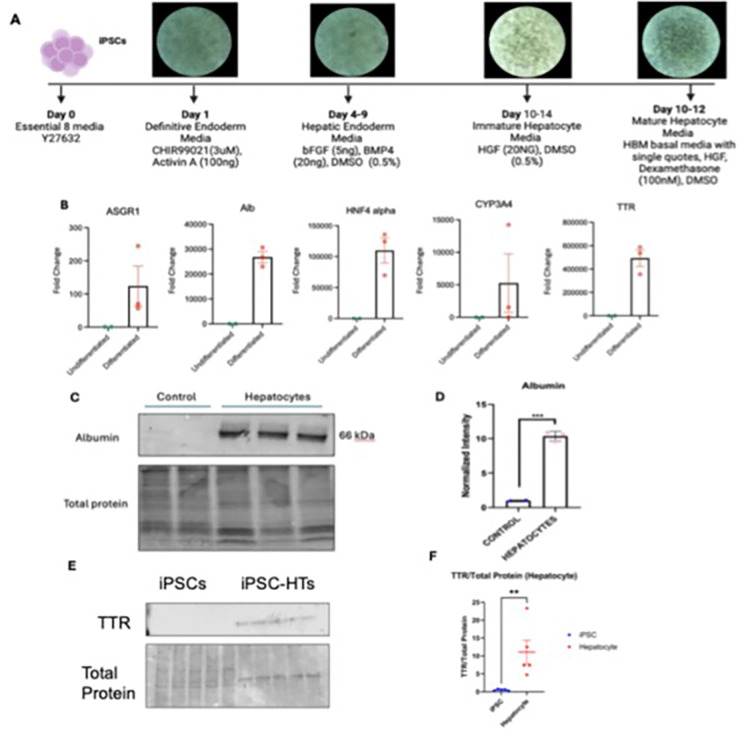
Differentiation of iPSCs into hepatocytes and characterization. **A)** Flowchart representing hepatic differentiation showing stepwise protocol along with images of the morphological changes observed across different stages. **B)** RT-qPCR data showed higher gene expression for ASGR1, Alb, HNF4 alpha, CYP3A4 and TTR in differentiated cells as compared to undifferentiated cells. **C)** Western blot data showing expression of hepatocyte marker albumin in hepatocytes as compared to control. **D)** Densitometric quantification of albumin protein expression. **E)**Western blot data showing expression of TTR in undifferentiated IPSCs compared to hepatocyte differentiated IPSCs. **F)** Densitometric quantification of TTR in hepatocytes compared to undifferentiated IPSCs.

hiPSCs were differentiated into hepatocytes through a four stage protocol: definitive endoderm (DE), hepatic endoderm (HE), immature hepatocyte (IMH), and mature hepatocyte (MH) as outlined in Fig. 3A. Activin A was used to induce DE, followed by basic fibroblast growth factor (bFGF) and bone morphogenetic protein 4 (BMP4) to promote hepatic specification. Expansion of immature hepatocytes was supported by hepatocyte growth factor (HGF), and final maturation was induced by dexamethasone and oncotatin M together with HGF in hepatic basal medium. Differentiated hepatocytes were harvested and characterized by RT-qPCR and western blot analysis. RT-qPCR demonstrated upregulation of asialoglycoprotein receptor 1 (ASGR1), albumin (ALB), hepatocyte nuclear factor 4-alpha (HNF4A), cytochrome P450 3A4 (CYP3A4), and TTR relative to undifferentiated hiPSC controls, which showed minimal or undetectable expression of these markers (Fig. 3B). Western blot analysis confirmed albumin protein expression in differentiated hepatocytes but not in undifferentiated hiPSCs (Figs. 3C–D). Similarly, TTR protein was detected in differentiated hepatocytes but was absent in undifferentiated controls (Figs. 3E–F). Together, these findings confirm successful hepatic differentiation and demonstrate that the hiPSC-derived hepatocytes have liver-specific gene expression and produce TTR.

### Characterization of cardiac organoids

**Figure 4:**
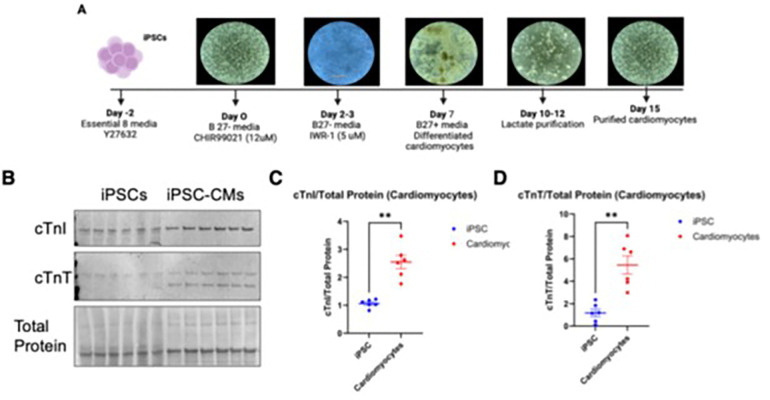
Cardiomyocytes differentiation and characterization. **A)** Flowchart showing the stepwise differentiation protocol along with images showing morphological changes in iPSCs and cardiomyocytes during different stages (scale bar = 390 μm). **B)**Western blot data showing expression of cardiomyocyte markers cardiac troponin T (cTnT) and cardiac troponin I (TnI) in differentiated cardiomyocytes as compared to controls (undifferentiated iPSCs). **C)** Densitometric quantification of cTnT and cTnI protein expression.

hiPSCs were differentiated into cardiomyocytes using established protocols, beginning with CHIR99021 (6 μM), followed by IWR-1 (5 μM) to augment Wnt signaling and drive cardiac specification. The differentiation and selection time is shown in Fig. 4A. Western blot analysis confirmed robust expression of cardiac troponin I (cTnI), cardiac troponin T (cTnT) in differentiated cardiomyocyte lysates, whereas undifferentiated hiPSC controls showed no detectable cTnT signal (Figs. 4B–D) suggesting successful generation of cardiac specific cells. These data verify efficient differentiation of hiPSCs into cardiomyocytes suitable for downstream organoid formation and co-culture studies.

### Microphysiological model of ATTR-CM

**Figure 5:**
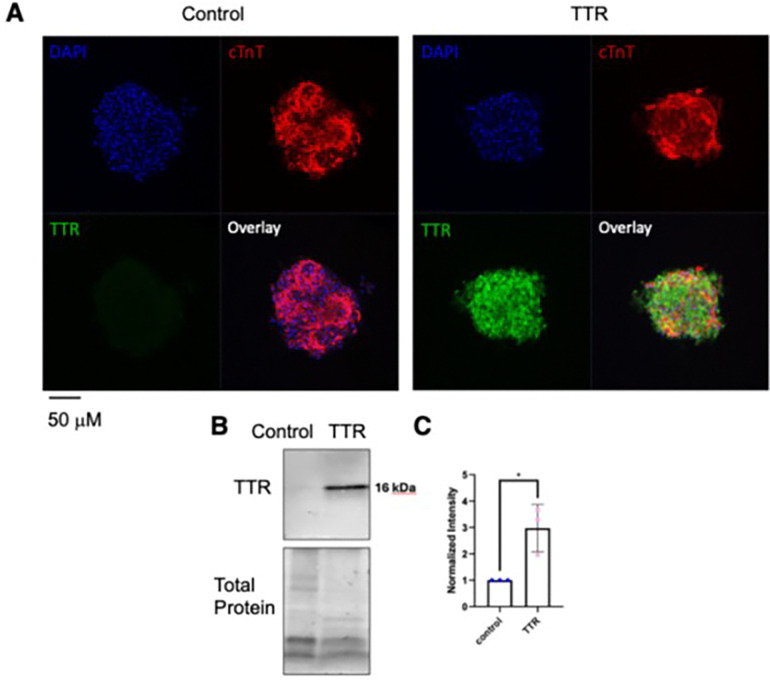
Uptake of TTR^V122I^ by cardiac organoids. **A)** Immunolabeling staining showing the presence of TTR (green) in cardiac organoids having TTR^V122I^ and in control conditions. DAPI (blue) indicates nuclei staining and cTnT (red) indicates cardiomyocyte marker. **B)** Western blot analyses show protein bands for TTR in cardiac organoids exposed to TTR^V122I^ as compared to controls. **C)** Densitometric quantification of transthyretin (TTR) protein expression.

To model ATTR-CM on the microfluidic platform, hepatic organoids generated from hiPSC-derived hepatocytes harboring the TTR^V122I^ mutation or control were seeded into one chamber, and cardiac organoids were loaded into the opposing chamber at a density of ~ 35–40 organoids per chamber. Co-cultures were maintained for 14 days with media exchanges occurring every 48 hours, after which cardiac organoids were harvested and analyzed for TTR uptake. Immunofluorescence staining revealed the presence of TTR within cardiac organoids cultured with TTR^V122I^ hepatic organoids, whereas control cardiac organoids exhibited no detectable TTR signal (Fig. 5A). Western blot analysis of cardiac organoid lysates confirmed the presence of monomeric TTR signal in mutant co-cultures but not in controls (Fig. 5B–C). Densitometric quantification demonstrated a significant increase in TTR protein in TTR^V122I^ exposed group (Fig. 3C). These results demonstrate that TTR^V122I^ synthesized and secreted by hepatic organoids successfully diffuse through microchannels and are subsequently taken up by cardiac organoids establishing a functional in-vitro model of the hepatic-cardiac molecular crosstalk pathway in ATTR-CM.

### Cytotoxic effects of TTR^V122I^ uptake in cardiac organoids

**Figure 6:**
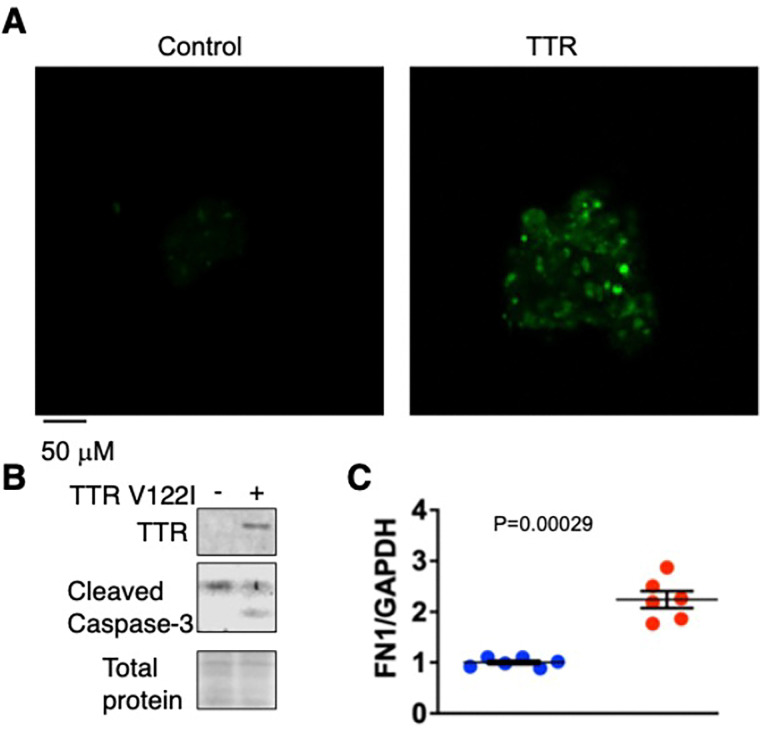
TTR^V122I^ uptake increases cytotoxicity and fibrotic markers in cardiac organoids. **A)** CELLROX assay reveals increased expression of ROS in cardiac organoids that took up TTR^V122I^. Cardiac organoids co-cultured in this system with TTR^V122I^ showed greater cell death, **(B)** western blot for cleaved caspase 3 and **(C)** and expression of fibronectin.

To evaluate downstream cellular consequences of TTR^V122I^ uptake, we assessed oxidative stress, apoptosis, and fibrotic gene expression in cardiac organoids. Reactive oxygen species (ROS) were measured using the CellROX green indicator. Confocal imaging revealed increased ROS signal in cardiac organoids cultured with TTR^V122I^ hepatic organoids compared with controls (Fig. 6A). In parallel, western blot analysis of apoptotic markers showed elevated levels of cleaved caspase-3 in TTR^V122I^ exposed cardiac organoids relative to controls (Fig. 6B). RT-qPCR demonstrated increased expression of the fibrotic marker fibronectin 1 (FN1) in the TTR^V122I^ group (Fig. 6C). Collectively, these findings indicate that cardiac organoids internalizing TTR^V122I^ exhibit key pathological features associated with ATTR-CM, including heightened oxidative stress, activation of apoptotic signaling, and upregulation of fibrotic gene expression.

### Functional impact of TTR^V122I^ uptake on engineered heart tissues (EHTs)

**Figure 7:**
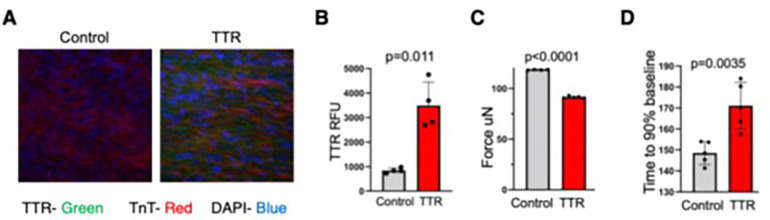
ATTR-CM EHTs take up TTR resulting in impaired function. **A)** Immunofluorescence staining of EHTs for TTR (green), cTnT (red), DAPI (blue) and **(B)** quantification indicating TTR^V122I^ uptake from the media in EHTs bathed in TTR^V122I^. **C)** EHTs cultured with TTR^V122I^ showed reduced force generation and **(D)** an increased time to return to 90% baseline.

To examine the functional impacts of TTR infiltration on cardiomyocyte dysfunction, we generated and engineered heart tissues (EHTs) from hiPSC-CMs and human cardiac fibroblasts on a mechanically loaded platform. After a three-week maturation period, EHTs were exposed to media containing monomeric TTR^V122I^ (5 μM) or control for 14 days. Confocal immunofluorescence imaging demonstrated robust TTR signal within TTR^V122I^-exposed EHTs that colocalized with cTnT-positive myocytes, whereas control tissues showed minimal or no TTR staining (Figs. 7A–B). Contractile performance was assessed using the MyoLab system. TTR^V122I^-treated EHTs exhibited reduced peak force (Fig. 7C) and prolonged relaxation time to 90% of baseline tension (Fig. 7D) compared to controls. These results indicate that EHTs take up TTR^V122I^ from the surrounding media and develop both systolic and diastolic dysfunction consistent with the contractile impairment observed in ATTR-CM patients.

## Discussion

Understanding the underlying mechanisms of ATTR-CM and evaluating potential therapeutic strategies has been hindered by the lack of models that faithfully recapitulate the physiological and pathological features of the disease. Access to ATTR-CM human myocardium remains limited, and transgenic mouse models require prolonged aging requirements while not reliably reproducing the pathological cardiac phenotype due to inconsistent amyloid deposition and fundamental interspecies differences.^12,13^ In vitro approaches utilizing exogenous recombinant TTR protein conditioned media, co-cultures that autonomously produce fibrils, and cardiomyocytes cultured on fibril substrates have provided valuable insight into TTR-mediated cardiotoxicity and oxidative stress accumulation.^13–16,18^ However, these approaches generally rely on manual TTR delivery rather than continuous secretion, are limited to two-dimensional tissue architectures, and are unable to provide functional readouts of tissue-level cardiac mechanical performance despite contractile dysfunction being a hallmark clinical feature of disease. We present two complementary human iPSC-based in vitro models that address these gaps.

ATTR-CM is distinct among cardiomyopathies in that the pathogenesis arises from TTR is secreted by the liver and deposits in the heart making the hepatic-cardiac axis central to recapitulate disease. Our microphysiological chip captures this axis in vitro by co-culturing hepatic and cardiac organoids in physically separate chambers connected by microchannels that allow continuous diffusion of soluble factors while preventing direct cellular migration. This design enables a complete pathogenic model of ATTR-CM in which hepatic organoids harboring the TTR^V122I^ mutation secrete amyloidogenic TTR that diffuses through the microchannels and is internalized by the cardiac organoids. The continuous nature of TTR fibril formation better recapitulates the chronic and progressive nature of TTR accumulation in patients as opposed to routine administration of bolus TTR.^13,18^ Cardiac organoids that internalized TTR^V122I^ exhibited increased oxidative stress, elevated cleaved caspase-3, and upregulation of fibronectin. Importantly, our models separated organoid design enables simultaneous interrogation of disease mechanisms at both the source and target tissues in disease. As such therapies that reduce hepatic TTR production and those that aim to provide cardiac protection can be evaluated for upstream and downstream effects. To our knowledge, this is the first in vitro platform that enables distinct dual-organ evaluation for ATTR-CM.

Contractile dysfunction is a defining clinical feature of ATTR-CM, presenting with diastolic impairment at early stages and progressing to reduced contractile reserve as disease advances.^1,3,30^ Despite this, no prior in vitro model enables the study of tissue-level cardiac mechanical performance in a human-based platform. Recent work has demonstrated that myocardial strips isolated from patient ATTR-CM myocardium produced reduced contractile force compared to non-failing controls providing direct evidence that amyloid deposition impairs systolic function.^31^ Our EHT platform complements this finding by recapitulating contractile dysfunction in a mechanically-loaded human iPSC-based system. EHTs exposed to monomeric TTR^V122I^ demonstrated amyloidogenic protein uptake followed by reduced peak force and prolonged relaxation mirroring the pathological clinical phenotype. Unlike the two-dimensional monolayer cultures used in most prior ATTR-CM models, the three-dimensional mechanically loaded EHT format enables direct assessment of contractile performance and can be further leveraged to investigate preload-dependent parameters such as the Frank-Starling response and systolic reserve, both of which are impaired in ATTR-CM patients but have yet to be studied in vitro.^1,13,31^

These two platforms are designed to be complementary. The microphysiological chip captures the inter-organ biology of ATTR-CM and is suited for evaluating upstream interventions that target hepatic production of TTR, while the EHT model captures the functional downstream consequences of TTR deposition and provides quantitative mechanical readouts. Together, they enable the study of the entire disease cascade from hepatic secretion to myocardial dysfunction in a human-based ATTR-CM in vitro platform. This is particularly relevant as current standards of care utilizing TTR silencers and protein stabilizers have demonstrated efficacy in slowing disease progression, but advancement towards heart failure remains a major clinical challenge.^6,11^ Together, these platforms provide a preclinical framework to investigate the mechanisms driving cardiac dysfunction in ATTR-CM and to evaluate novel therapeutic strategies, including combination approaches that target both TTR production and myocardial function restoration.

## Limitations

While this study establishes new platforms to facilitate investigations into ATTR-CM, several limitations should be noted. It is thought that multiple hits are required for someone to develop ATTR-CM; these co-factors remain largely unknown, making comprehensive modeling challenging. Our systems focus on the hepatic-cardiac axis and do not yet incorporate additional components such as immune cells, endothelium/vasculature, or autonomic innervation, which may modulate amyloid deposition and remodeling. As with many iPSC-derived models, cardiomyocyte maturation may be incomplete, potentially affecting electrophysiological and contractile phenotypes. We modeled the TTR^V122I^ variant, but variant specific effects may differ and while we think our findings will generalize to other variants, this is not tested here. Finally, we did not test current standard of care therapies for ATTR-CM in these model systems. The goal of this study was to establish and validate in vitro platforms that recapitulate the biochemical, cellular, and functional characteristics of ATTR-CM. Evaluation of TTR-directed and cardiac-directed therapies, alone and in combination, will be the focus of subsequent studies.

## Conclusions

These complementary human-based models recapitulate central features of ATTR-CM and enable mechanistic interrogation across molecular, cellular, and functional levels. They provide translational platforms to test therapeutic strategies aimed at reducing amyloid burden and improving cardiac performance, including stabilizers, silencers, aggregation inhibitors, anti-fibrotic agents, and contractility-modulating approaches. Future work will leverage these systems for preclinical screening, variant-specific studies, and integration of additional cell types to further enhance the physiological fidelity and clinical relevance of these in vitro ATTR-CM models.

## Data Availability

All data supporting the findings of this study and protocols used in this study are available within the article. Any additional requests for information can be directed to and will be fulfilled by the corresponding author upon reasonable request sent to mranek1@jh.edu. Source data are provided with this manuscript.
